# Knowledge, Attitude, and Practice of Evidence-Based Medicine among Emergency Doctors in Kelantan, Malaysia

**DOI:** 10.3390/ijerph182111297

**Published:** 2021-10-27

**Authors:** Mohd Ghouse Ahmad Ghaus, Tuan Hairulnizam Tuan Kamauzaman, Mohd Noor Norhayati

**Affiliations:** 1 Department of Emergency Medicine, School of Medical Sciences, Health Campus, Universiti Sains Malaysia, Kubang Kerian 16150, Kelantan, Malaysia; ahmad.ghaus.786@gmail.com (M.G.A.G.); hairulnizam@usm.my (T.H.T.K.); 2 Department of Family Medicine, School of Medical Sciences, Health Campus, Universiti Sains Malaysia, Kubang Kerian 16150, Kelantan, Malaysia

**Keywords:** evidence-based medicine, emergency medicine, knowledge, positive, practice

## Abstract

This study aimed to determine the prevalence of high levels of knowledge, positive attitude, and good practice on evidence-based medicine (EBM) and identify the associated factors for practice score on EBM among emergency medicine doctors in Kelantan, Malaysia. This cross-sectional study was conducted in government hospitals in Kelantan. The data were collected from 200 emergency physicians and medical officers in the emergency department using the Noor Evidence-Based Medicine Questionnaire. Simple and general linear regressions analyses using SPSS were performed. A total of 183 responded, making a response rate of 91.5%. Of them, 49.7% had a high level of knowledge, 39.9% had a positive attitude and 2.1% had good practice. Sex, race, the average number of patients seen per day, internet access in workplace, having online quick reference application, and attitude towards EBM were significantly associated with EBM practice scores. It is recommended that appropriate authorities provide emergency doctors with broader access to evidence resources. EBM skill training should be enhanced in the current medical school curriculums.

## 1. Introduction

Evidence-based medicine (EBM) is defined as “the conscientious, explicit and judicious use of current best evidence in making decisions about the care of individual patients” [1] (p. 71). The EBM is a paradigm for clinical practice involves integrating the best available evidence with individual clinical expertise with consideration of individual patient’s right and preferences in clinical decision making [2]. The five basic steps in applying EBM include defining the problem, searching for resources and databases, critically evaluating information, obtaining valid evidence, and evaluating the usefulness and effectiveness of the evidence [3].

One of the most significant principles in EBM is the hierarchy in validating evidence based on which judgments are made, which indicates that it is critical to evaluate the value of evidence before making decisions. The evidence is ranked in the hierarchy based on their likelihood of bias. Evidence obtained by meta-analysis of several randomized controlled trials is highest in the hierarchy since they are designed to be unbiased and have a lower likelihood of systematic mistakes. A case series or expert opinion is frequently skewed by the author’s experience or opinions, and confounding factors are not controlled for [4].

Clinicians must have solid communication skills, as well as an ethical awareness and understanding of cultural and societal impacts on patient encounters. These characteristics aid physicians in gaining a better understanding of their patients’ needs and preferences in order to better manage their illnesses and select suitable interventions. Establishing professional standards can be aided by guidelines and credible study summaries that are based on solid research data [5]. By bridging the gap between best practice and standard care and reducing dangerous and ineffective treatments, evidence-based clinical practice saves money and improves healthcare quality [6]. The goal of EBM is to enhance the quality of care by supporting good practices and encouraging clinicians to try new scientific methods and abandon ineffective ones [2].

In a systemic review, many physicians reported having poor EBM knowledge and skills. Yet, they have a positive attitude towards implementing EBM in their daily practice. [7]. Another systemic reported lack of suitable facilities, low motivation, negative attitude towards EBM, lack of confidence in research results and lack of training in EBM. Resistance to change was also identified as a barrier in EBM [8].

General practitioners in the United Kingdom welcome EBM and agree that it improves patient care. However, they had a low level of awareness regarding extracting journals, reviews, and databases [5]. The knowledge of doctors in Saudi Arabia was considered acceptable. However, the gap between understanding and practice of EBM was due to time and accessibility of resources limitations [9]. A study among Norwegian physicians reported limited knowledge of EBM but a positive attitude towards implementing EBM in clinical practices. They agreed that EBM helps physicians improve practice and patients’ care. Instead, most physicians would consult their colleagues rather than searching for evidence-based resources [10].

Over recent years, EBM has created an impact on emergency medicine worldwide. It is associated with a high workload but, at the same time, is required to uphold the quality of healthcare. Emergency doctors and paramedics are on the front line of emergency care services, require the latest updates in disease management, and must keep patients’ best interests in mind. There have been few studies conducted in Malaysia on EBM. However, these are limited by the study population size and may not be representative [11,12].

Emergency doctors work in resuscitation areas that are both time-critical and information-sparse. To the best of our knowledge, no study evaluating the application of EBM has been conducted among emergency doctors. To address this gap, this study aimed to determine the prevalence of high level of knowledge, positive attitude, and good practice on EBM and identify the associated factors for practice score on EBM among emergency medicine doctors in Kelantan. We believe this study will help promote EBM among emergency doctors and provide data for appropriate authorities to provide emergency doctors with broader access to EBM resources.

## 2. Materials and Methods

### 2.1. Population and Sample

This is a cross-sectional study among emergency doctors in Kelantan. It is a state located in the north-eastern corner of Malaysia with an estimated population of 2,001,000. The state has 10 hospitals with approximately 250 emergency medical officers working under the supervision of 30 emergency physicians in the emergency department. This study includes emergency medical officers and emergency physicians in district hospitals and general hospitals. It excluded house officers and those working in private hospitals or retired from practice.

Simple random sampling was used. The sample size was calculated based on a single proportion formula [13] for the prevalence of a high level of knowledge of EBM. Assuming the precision of 0.05 with a 95% confidence interval and nonresponse rate of 10%, a sample of 200 emergency doctors was needed.

### 2.2. Research Tool

The questionnaire consists of five sections. Section 1 includes the sociodemographic data of the population such as age, sex, race, marital status, qualification, place of practice, and years of clinical experience. Section 2 consists of environmental factors pertinent to the study, such as the average number of patients seen in a day, internet in the workplace, accessibility to subscribed online databases, online mobile applications, and supportive colleagues.

Section 3, Section 4 and Section 5 utilized the validated Noor Evidence-Based Medicine Questionnaire on knowledge, attitude, and practice among primary care practitioners [14,15]. It consists of 15 knowledge, 17 attitude, and 11 practice items on EBM. The knowledge and attitude items are assessed based on a five-point Likert scale of “Strongly Agree” = 5, “Agree” = 4, “Neutral” = 3, “Disagree” = 2, “Strongly Disagree” = 1; while the practice items are assessed based on five-point Likert scale of “Always” = 5, “Often” = 4, “Sometimes” = 3, “Seldom” = 2, “Never” = 1 [15]. The psychometric properties for the knowledge domain of EBM were also analysed based on the item-response theory for the Rasch model. The rating scale diagnostics suggest collapsing of category 1 (observed count = 15, 1%; outfit MnSq = 1.91) and category 5 (observed count = 221, 16%; outfit MnSq = 1.01) with sensible adjacent category categories. A three-point Likert scale of “Correct = 3”, “Not sure” = 2, “Wrong” = 1, with reverse scoring for negative-worded items, was suggested [14].

Total scores were calculated for each knowledge, attitude, and practice domain. Each total raw score was transformed into a “percent score” and categorized based on Bloom’s cut-off point [16]. Scores less than 59% are determined to have a low, negative, and poor level of knowledge, attitude, and practice. Scores within 60–80% were equated with a moderate, neutral, and fair level of knowledge, attitude and practice. Scores more than 81% are determined to have a high, positive, and good level of knowledge, attitude, and practice [17].

### 2.3. Data Collection

The approval for this research was obtained from the human research ethics committee of Universiti Sains Malaysia (USM/JEPeM/1909514) and the medical research ethics committee of the ministry of health (NMRR-19-2502-50090). Data collection was conducted from February‒May 2020. The researcher made appointments with the prospective participants at their respective facilities, explained the study, and distributed the informed consent forms. When the participants understood and consented to join the study, they were given the self-administered questionnaire. They were free to ask the researcher if they encountered problems while answering it. The time estimated to complete the case report form was 20 min. The questionnaires were checked for completeness, and the participants were thanked for their cooperation.

### 2.4. Data Entry and Analyses

The data were entered and analyzed using SPSS version 26 (SPSS Inc., Chicago, IL, USA, 2019). Before analysis, the data were checked and cleaned. Then, descriptive analyses were conducted to define a high level of knowledge, a positive attitude, and a good practice of EBM. Simple and general linear regression analyses were used to determine the factors associated with EBM practice scores.

## 3. Results

A total of 200 respondents were invited. However, only 183 responded, making a response rate of 91.5%. The nonrespondents did not complete their questionnaires. The median age of the responders was 31. The majority of the respondents (*n* = 156, 48.6%) were of Malay ethnicity, with 94 (51.4%) females. The percentage of respondents who were married at the time of the survey was 53%, while the rest were single. Bachelor’s degree holders accounted for 153 (83.6%) of the respondents, followed by Master’s degree holders 30 (16.4%). The majority of responders (*n* = 129, 70.5%) worked in a general hospital. Most of the respondents had internet access in the workplace (*n* = 175, 95.6%) with 152 (83.1%) of them had online quick reference application. Continuous medical education was accessible in the majority of respondent workplaces (*n* = 162, or 88.5%). Sociodemographic profiles of the 183 emergency doctors are shown (Table 1).

Ninety-one respondents (49.7%) were classified as having a high level of knowledge. It was followed by moderate (47.5%, *n* = 87) and low (2.8%, *n* = 5) levels of knowledge. The majority of the respondents only knew two components of EBM: critically appraising research findings (90.2%) and patient care (56.8%), whereas the third component, clinical expertise, was incorrectly answered (54.6%). When it came to the use of EBM in clinical decision making, the majority of respondents (79.2%) knew about using the PICO format to create a good clinical question, 90.7% knew that EBM can be used if there is a doubt in clinical management, and 90.2% agreed that improved access to summaries evidence would encourage evidence-based practice. However, only 3.3% of respondents were aware that EBM encourages self-directed learning (see Appendix A Table A1 for responses to knowledge items related to EBM).

Seventy-three respondents (39.9%) were classified as having a positive attitude towards EBM. It was followed by neutral (54.6%, *n* = 100) and negative (5.5%, *n* = 10) attitudes towards EBM. EBM is a threat to good clinical practice, according to the majority of our respondents (97.8%). Despite this, the majority of them believe EBM will improve patient outcomes (95.6%) and are eager to learn EBM if given the chance (95.1%). Moreover, half of the respondents (51.9%) believed that research findings are critical in their day-to-day patient treatment. The majority of respondents strongly agreed that database access is critical for getting EBM journals (45.9%) (see Appendix A Table A2 for attitude items towards EBM and possible response).

Four respondents (2.1%) were classified as having good practice. Most respondents had poor (74.9%, *n* = 137) followed by moderate (23.0%, *n* = 42) levels of practice. Only 3.3% of those respondents never attend continuing medical education to stay up to date on EBM. When it came to having no time to study EBM, the majority of our respondents (44.8%) selected “Sometimes.” For systemic review, the vast majority of responders (98.4%) use multiple search engines (see Appendix A Table A3 for practices related to EBM and possible response).

General linear regression showed that sex, ethnicity, the average number of patients seen per day, availability of subscribed online databases in the workplace, having online quick reference applications, and neutral attitude towards EBM were significantly associated with EBM practice scores (Table 2).

## 4. Discussion

In this study, the respondents’ knowledge of EBM was mainly high or moderate. In comparison to a survey conducted among primary care doctors in Selangor, Malaysia, using a similar research instrument, it was found that 60.9% of the respondents had a moderate level of knowledge, whereas 6.2% had a low level of knowledge [17].

Statistical terms in EBM have been known to cause difficulty in applying the evidence-based practice in this study. An almost similar finding was found among the primary care in Selangor [17]. These findings were also supported further by a study in Melaka, which found that more than half of the respondents did not understand the terms used, i.e., number needed to treat, meta-analysis, odds ratio, and confidence interval. In comparison, more than half of the respondents have some understanding of terms like relative risk and absolute risk [12]. However, studies from other countries have different findings. For example, in Sri Lanka, less than 38% of the medical officers understood some statistical phrases like systemic review and meta-analysis [5]. On the other hand, in a study done among doctors in England, one-third of them could understand the statistical terms and able to explain to others the meaning of the statistical terms [18].

In our study, most of the respondents understood that EBM involves critically appraising research findings to make relevant clinical decisions. Critical appraising is a systemic method to evaluate the evidence for its validity and clinical usefulness systemically. It is a crucial step because it lets the clinician decide whether an article can be relied on to provide helpful guidance or information. Individuals without research expertise can master critical appraisal, which entails learning how to ask a few key questions about the validity of the evidence and its relevance to a particular patient or group of patients. These fundamentals can be trained within a few hours in tutorials, workshops or interactive lectures [19]. A study of 1080 Hungarian medical students found that those who had EBM training rated their skills in critical appraising significantly better than students who did not receive EBM training [20]. In a French study involving 397 health care professionals, the authors found that lack of skills for critical appraisal of studies was perceived as a barrier in EBM among 21.7% of respondents [21].

Almost half of the respondents in this study answered wrongly regarding EBM focuses on the best current available research without considering clinical experience. In comparison, in a three-point Likert scale survey, half of the physicians perceived clinical experience or physicians skills as “Quite” important component of EBM [22]. Evidence-based medicine is defined as a systematic approach to clinical problem solving, which allows the integration of the best available research evidence with clinical expertise and patient values. Here, we need to emphasize that clinical expertise is one of the vital parts in EBM. The skills that physicians acquired during years of clinical practice and clinical experience is a necessary and indispensable part of what makes a good doctor [4].

A similar outcome was found in our study with regards to making a decision with consideration of patient preferences. Nearly half of the respondents answered wrongly in which they considered that there is no need for patients’ preferences in making a clinical decision. In comparison to a study, 61% of the participants perceived patients’ will as “Quite” important component in EBM [22]. This can be concluded that our respondents were not aware of another core component of EBM, including patient values and expectations [4].

In the current study, half of the respondents understood that EBM is suitable for making decisions about patients’ care and policymaking. One of the World Health Organization’s goals for the 21st century is producing and expanding knowledge for evidence-based policy and implementation [23]. EBM is often portrayed as the “model” for evidence-based policymaking. There are three advantages to assist in policymaking. Firstly, EBM is more transparent about the processes and structures used to find and use evidence. Secondly, EBM considers how to balance evidence and other interests (i.e., clinician clinical experiences and patients’ expectation). Third, it helps to provide a forum for shared discussion and sense-making among the researchers, public, and stakeholders [24]. In a descriptive study among pharmacists in Kuwait, the authors concluded that higher organizations should develop policies to practice EBM to organize, standardize, and facilitate clinical framework [25].

In this study, the majority of the respondents knew that EBM has four essential components structured in the PICO format, which will facilitate in making good clinical questions. The previous study also had a similar response, which was 81.3% [17]. A focused clinical question containing PICO elements is believed to be the key to efficiently finding high-quality evidence and clinical decision [26]. The PICO framework was initially developed for therapy questions and later extended to all types of clinical questions. It is a tool that has been tested, and it is adequate and suitable as a knowledge representation for clinical questions [27].

In our study, more than half of the respondents answered correctly regarding meta-analysis ranked higher in the level of evidence when compared to case-control studies. This item is made to test the knowledge of the respondents regarding the strengths of various study designs. Almost all knew that practicing EBM improves clinicians’ understanding of research methodology. In EBM, evidence refers to what is proved by studies conducted according to the best research design. For example, a study design which is a randomized controlled, double-blind, placebo-controlled trial conducted with a homogenous patient group and followed up, provide the least risk of bias and the strongest evidence. On the contrary, a case report or an expert opinion is considered a weak level of evidence because it has a high probability of bias [28].

Eighty-two percent of the respondents are not aware that EBM does promote self-directed learning. Self-directed learning is recognized as an important tool in the medical profession as medicine, and its practice changes so rapidly as time goes. It is a lifelong process. The framework of self-directed learning and EBM is not widely differing. Self-directed learning is a process in which individuals take the initiative, with or without others’ help, in diagnosing their learning needs, formulating learning goals, identifying human and material resources for learning, choosing and implementing appropriate learning strategies and evaluating learning outcomes. These steps are near the PICO framework mentioned [29]. In EBM practice, clinicians can ask effective clinical questions, acquire information through emerging research, appraise its quality and relevance based on the research methodology, apply evidence to practice, and assess its impact on patient and practice [30]

The majority of our respondents knew that the application of evidence-based practice is cost-effective to the healthcare system. It was found that evidence-based treatment in non-small-cell carcinoma resulted in an average cost savings of 35% over 12 months [31]. As for general physicians, the National Health Service England has already issued GP prescription guidance, which was evidence-based interventions. The challenge here is for the GP to stop practicing ineffective or low-value interventions. Higher quality medical care may even happen to be less expensive [32].

The EBM can be practiced in situations where there is doubt about any aspect of clinical management. The majority of our respondents acknowledged this fact. Three main elements need to be fulfilled when there is a doubt in clinical practice and applying the evidence. First, the evidence must be good evidence that each test or procedure recommended is medically effective in reducing morbidity or mortality. Secondly, the medical benefits must outweigh the risks; thirdly, each test or procedure’s cost must be reasonable compared to its expected benefits. The recommended actions must be practical and feasible [33].

In our study, about 40% of the respondents have a positive attitude towards EBM. Compared to another local survey among primary care doctors, 12% of the respondents have a positive attitude towards EBM [17]. It suggests that the attitude towards EBM may vary among subspecialties. A study in Wuhan, China, also found that the physicians’ specialties were significantly associated with their attitudes towards EBM [22]. In Saudi Arabia, different specialties scored differently towards the attitudes, in which surgeons scored the lowest attitude score while the pediatricians scored the highest [34]. The variation among the subspecialties can be due to the incorporation of EBM training in the residency syllabus. In a survey in the United States, the authors found that only one-third of the residency programs offered EBM curricula that targeted EBM skills [35].

The majority of our respondents agree that EBM is a threat to good clinical practice. These findings can be supported by a qualitative study in Klang Valley that was conducted among primary care practitioners. The authors found that most of the doctors considered EBM a threat to clinical practice. This negative perception increases the resistance to accept EBM [11].

Despite the unfavorable attitude towards EBM, most of our respondents perceived that EBM would improve patients’ health outcomes and learn EBM if given the opportunity. It agrees with findings in a study in Sri Lanka and Egypt where more than 80% of the doctors agree that EBM will improve patients’ outcomes [2,5]. However, a systemic review found no evidence suggesting that EBM training will improve patients’ outcomes. Yet, investigating the relationship between patient outcomes with EBM training is difficult because the outcomes may be related to many different factors in the clinical context. Therefore, it isn’t easy to measure the direct contribution of EBM among these many factors [36].

We also found that most of the respondents agreed that research findings were essential in their day-to-day management of patients. It is also similar to primary care physicians in Selangor [17] and Jordan [37], in which 68% and 67% of the respondents, respectively, agreed that research findings are useful in their daily practices. Among our respondents, only 68% disagree that EBM has limited value in the management of emergency care. This result is comparatively better than other two studies conducted in Selangor [17] and Jordan [37].

We found that almost half of our respondents were neutral towards the notion that years of clinical experience are more valuable than EBM. This finding was also mirrored among the primary care physicians in Selangor. Intuition plays a vital role in clinical practice. Although it has varied definitions, the recurring element was that intuition has its origins from personal clinical experience. In a qualitative study among 15 general practitioners, some of them described themselves as EBM practitioners includes intuition in clinical their clinical decision making. Thus, EBM and intuition are not two opposing forces instead, both are complementary to each other [38].

Most of the respondents feel that having access to databases is vital in obtaining EBM journals. PubMed (65.3%) was the most widely visited database among local primary care practitioners, while UpToDate was only accessed by one-third of those who responded. [17]. In a cross-over randomized trial held in Tehran, the authors found that for first-time users accessing a database to answer a clinical question, using UpToDate compared to PubMed can lead to a higher proportion of relevant answers within a shorter time and higher clinician satisfaction [39]. It varied from a study conducted in Egypt in which 61.3% of the physicians used PubMed in clinical decision-making rather than UpToDate, which was used by only 19.2% of the physicians [2].

In the current study, we observed that the respondents’ good practice on EBM was low. Similarly, among the primary care doctors in Selangor, their prevalence of good practice on EBM was only 0.4% [17]. Likewise, less than 10% of the Jordanian primary care physicians applied EBM in their daily clinical practices [37]. In France, only a minority of health professionals (14.2%), including physicians, nurses, and pharmacists, admitted to using EBM regularly in their daily practice [21]. The barriers for good practice identified from the previous studies were poor attitude towards EBM, lack of access to information, lack of time, and lack of critical appraisal skills.

More than 90% of the respondents to this current study joined CME to get updated on EBM. This is also similar to findings among primary care physicians in Selangor, in which 94.2% of them receive updates regarding EBM by joining CME. This good response towards CME is possibly due to the compulsory requirement set by the Malaysian medical council to renew the annual physician practice license [40]. Lower prevalence was found in a study in Wuhan, China, in which 61.8% of the participants use CME as the primary channel to learn about EBM in comparison to school education, hardcopy journals, internet, colleagues and advanced training [22].

Clinical practice guidelines (CPG) are defined as statements that comprise recommendations intended to optimize patient care that are informed by a systematic review of evidence and an assessment of the benefits and harms of alternative care options. In Malaysia, the CPG development group and review group include senior consultants in public healthcare facilities and private consultants [41]. The majority of our respondents are not involved directly in the development of CPG. This is because 83% of our respondents are emergency medical officers, and only a minority are emergency physicians.

The majority of our respondents chose “Sometimes” on having no time to study EBM. In a study conducted in Egypt, 60% of the physicians recognized that lack of time as a barrier in practicing EBM [2]. This is further supported by studies done locally and internationally that agree that lack of time is one of the barriers in practicing EBM [7,12,42].

In the current study, 98% of the respondents used multiple search engines for literature searches. This finding is further supported by a study done in Egypt, in which 87.6% of the participants used the Internet for literature searches [2]. However, in this current study, we do not test the respondents’ competency on online searches. In Oman, from a self-administered survey among the medical residents, about 57% of the respondents were competent users of search engines compared to 25% of the respondents who rated their skills as neutral [43].

In the present study, most of the participants (32.8%) chose “Often” when reporting the sharing knowledge of EBM among colleagues in the workplace. Sharing knowledge about EBM among colleagues can be considered as a medium. Similar findings were reported among physicians in Wuhan in which 34.4% of the participants do get their knowledge of EBM from colleagues [22]. Despite this, colleagues’ attitudes can be a barrier to practicing EBM. This is reported by Abdeel-Kareem, in which 47% of the participants perceived that their colleagues’ attitudes are a barrier to EBM practice [2]. In a systemic review regarding EBM, the authors found that the majority of the physicians commonly refer to their colleagues or expert in the fields in an attempt to answer their clinical questions [7].

The majority of our respondents rarely or never had time to study EBM. For example, in a study conducted in Egypt, 60% of the physicians recognized that lack of time was a barrier to practicing EBM [2]. It is further supported by studies done locally and internationally that agree that lack of personal time to study EBM is one of the barriers in practicing EBM [7,17,21,22,44].

In the present study, most participants frequently shared EBM knowledge of EBM among colleagues in the workplace. Therefore, sharing knowledge about EBM among colleagues can be considered as a medium. Similar findings were reported among physicians in Wuhan, in which 34.4% of the participants do get their knowledge of EBM from colleagues [22]. Despite this, colleagues’ attitudes can be a barrier to practicing EBM. This was reported by Abdeel-Kareem’s study: 47% of the participants perceived that their colleagues’ attitude is a barrier to EBM practice [2]. In a systemic review regarding EBM, the authors found that most physicians commonly refer to their colleagues or to experts in their field to answer their clinical questions [7].

We also determined that sex, race, the average number of patients seen in a day, availability of subscribing to an online database in the workplace, quick online references, and a neutral attitude have been significantly associated with the practice of EBM at the workplace. Previous research found that race, attitude, length of work experience and quick access to online reference applications were significantly associated with the practice of EBM [17]. We found that female healthcare providers have relatively poor practice towards EBM compared to their male counterparts. The effect of gender on the practice of EBM has also been explored in previous studies. In a survey conducted among healthcare providers in Ethiopia, the authors found that male participants were more likely to have more knowledge of EBM than females [6].

On the contrary, another study found no association between knowledge of EBM and sex [20]. With reference to our study, there was no association between having good knowledge of EBM and good practice of EBM. Similar results were also found in a study in France, in which there was no gender difference in terms of the depth of EBM knowledge and EBM practice [21].

In this current study, we found that being Malay was associated with emergency doctors practicing EBM. That is further supported by a survey among primary care physicians in Selangor, which found that non-Malay physicians had good practice of EBM [17]. However, no other local studies have explored the relationship between race and practice of EBM [11,12,45]. Furthermore, up to our knowledge, there are also no international studies that examine the relationship between race and evidence-based practice, mainly because not many countries are as multiracial as Malaysia.

We also found that quick online references in the workplaces were associated with the practice of EBM. A study in France found that the absence of availability and access to information have been perceived as obstacles in EBM practice [21]. In another study in Kuwait, among pharmacists, the authors found that 75% identified that access to online EBM resources is a barrier in EBM practice [25]. EBM libraries should be easy to use, contain up-to-date information, and have electronic databases. Cochrane Library, Best Evidence, UpToDate, Evidence-Based Medicine Review, and Medscape have these features [28].

We discovered that the average number of patients seen per day was related to the level of EBM practice, particularly in the groups of 10–20 and 21–30 patients. This finding was contrary to a survey among primary care physicians in Selangor which found no relevance between the average number of patients seen per day to the level of EBM practice [17]. Contrarily, patient overload has been reported as a barrier in EBM practice in different world regions. Sixty-eighty percent of the participants in an Egyptian study found that patient overload was reported as a barrier to EBM [2]. It is further supported by a study in France that 26.1% of the participants perceived lack of time as an obstacle in EBM practice [21]. In Wuhan, China, 32.5% of physicians perceived lack of time as a barrier in implementing EBM practice [22]. In a systemic review of 56 studies regarding EBM, patient overload was perceived as one barrier in EBM practice [7].

In this study, having a neutral attitude among physicians was significantly associated with the practice of EBM. This finding is different to that of another local study in which having a negative attitude was significantly associated with the practice of EBM [17]. The poor attitude was a barrier in practicing EBM [7]. Besides that, a few studies also reported that colleagues’ attitudes were a barrier to implementing EBM [2,46]. Factors that affect attitudes to EBM practice were gender, clinical specialty, previous medical qualifications, and previous EBM training [2].

Historically, research has demonstrated that internet accessibility has been a barrier to EBM practice [5]. This is a shift from recent years when internet connectivity was not seen as a significant obstacle to EBM practice, as supported by this and other studies [2,17].

This study has several limitations. The emergency doctors have a high patient workload; therefore, they had a limited time to answer the questionnaires, leading to nonresponse bias. We are also aware that the questionnaire relied on the self-rated assessment of knowledge and beliefs. As a result, research participants might have felt pressured into completing the questionnaire or might have been unwilling to divulge their knowledge and skill deficiencies.

## 5. Conclusions

Although most emergency doctors have good knowledge and a positive attitude towards EBM, they have a low EBM practice. Factors associated with the poor practice of EBM were higher patient volume, ethnicity, poor access to subscribed databases, unavailability of access to the online application, and a neutral attitude towards EBM. The gap and barriers recognized in this study can serve as baseline data to design effective interventions to improve emergency doctors’ knowledge, attitude, and practice. This includes giving them more access to evidence resources and boosting awareness of the importance of incorporating EBM skill training into ongoing medical education, and improving current medical school curricula.

Decision-makers should also include strategies for fostering change among emergency doctors to become more EBM-oriented. It is high time to establish a Malaysian EBM board to aid in disseminating EBM concepts, methodologies, and practices among Malaysian doctors.

## Figures and Tables

**Table 1:** Characteristics of the study respondents (*n* = 183).

Variables	Median (IQR ^a^)	*n* (%)
Age (year)	31 (4)	
Experience in current workplace (year)	4 (5)	
Sex		
Male		89 (48.6)
Female		94 (51.4)
Ethnicity		
Malay		156 (85.2)
Non-Malay		27 (14.8)
Marital status		
Single		86 (47)
Married		97 (53)
Highest academic qualification		
Bachelor		153 (83.6)
Master		30 (16.4)
Place of practice		
General Hospital		129 (70.5)
District Hospital		54 (29.5)
Average number of patients seen per day		
<10		21 (11.5)
10−20		97 (53.0)
21−30		32 (17.5)
>30		33 (18.0)
Availability of provided internet access in workplace		
Yes		175 (95.6)
No		8 (4.4)
Availability of subscribed online databases in workplace		
Yes		124 (67.8)
No		59 (32.2)
Presence of online quick reference application		
Yes		152 (83.1)
No		31 (16.9)
Presence of continuous medical education in workplace		
Yes		162 (88.5)
No		21 (11.5)

Note: Description is based on Noor Evidence-Based Medicine Questionnaire [15]. ^a^ Interquartile range.

**Table 2:** Factors associated with EBM practice in emergency doctors using simple and general linear regression analyses (*n* = 183).

Variables	Simple Linear Regression			General Linear Regression ^e^		
	Crude reg. coef. ^a^ (95% CI ^b^)	*t* Stat ^c^	*p* Value	Adj. reg. coef ^d^ (95% CI ^b^)	*t* Stat ^c^	*p* Value
Age (year)	1.1 (0.36, 1.79)	2.99	0.003			
Emergency department experience (year)	0.9 (0.15, 1.59)	2.38	0.020			
Sex						
Male						
Female	−5.7 (−9.89, 1.45)	−2.65	0.009	−6.3 (−9.90, −2.64)	−3.41	0.001
Ethnicity						
Malay						
Non-Malay	11.1 (5.29, 16.96)	3.76	<0.001	8.5 (3.62, 13.51)	3.42	0.001
Marital status						
Single						
Married	0.6 (−3.75, 4.86)	0.26	0.798			
Highest academic qualification						
MBBS/MD/MBChB						
Specialist	5.3 (−0.50, 11.00)	1.80	0.730			
Place of practice						
General hospital						
District hospital	−7.4 (−11.97, −2.80)	−3.18	<0.001			
Average number of patients per day						
<10						
10–20	5.6 (−1.11, 12.48)	1.65	0.101			
21–30	8.5 (0.59, 16.45)	2.12	0.035	4.9 (0.20, 9.52)	2.06	0.041
>30	13.4 (5.53, 21.29)	3.36	0.001	8.0 (3.43, 12.60)	3.45	0.001
Internet access in workplace						
Yes						
No	−10.7 (−21.05, −0.27)	−2.02	0.044			
Subscribed online databases in workplace						
Yes						
No	−9.9 (−14.36, −5.64)	−4.43	<0.001	−6.8 (−10.95, −2.67)	−3.25	0.001
Online quick reference application						
Yes						
No	−11.7 (−17.14, −6.2)	−4.22	<0.001	−9.2 (−14.22, −4.19)	−3.62	<0.001
Continuous medical education						
Yes						
No	−7.8 (−14.46, −1.17)	−2.32	0.021			
Knowledge categories						
High						
Moderate	−4.0 (−8.34, 0.30)	−1.83	0.068			
Low	3.6 (−9.68, 16.80)	0.53	0.596			
Attitude catgories						
Positive						
Neutral	−9.6 (−13.88, −5.38)	−4.47	<0.001	−9.4 (−13.00, −5.86)	−5.21	<0.001
Poor	−2.8 (−12.08, 6.55)	−0.59	0.559			

Note: Variable is based on Noor Evidence-Based Medicine Questionnaire [15]. ^a^ Crude regression coefficient; ^b^ confidence interval; ^c^ *t* statistic; ^d^ adjusted regression coefficient; ^e^ general linear regression (R^2^ = 0.388; no significant interaction; no multicollinearity problem; model assumptions met).

## Appendix A

**Table A1:** Responses to Knowledge on Evidence-Based Medicine.

Item	Description	Correct n (%)	Unsure n (%)	Wrong n (%)
K1	Evidence-based medicine involves the process of critically appraising research findings as the basis for clinical decisions.	165 (90.2)	14 (7.7)	4 (2.2)
K2	Evidence-based medicine focuses on the best current available research without considering clinical experience.	41 (22.4)	42 (23.0)	100 (54.6)
K3	Evidence-based medicine is suitable for making decisions about care of patients rather than for policy making.	104 (56.8)	43 (23.5)	36 (19.7)
K4	Patients’ preferences should be considered first than clinicians’ preferences in making clinical decisions.	53 (29.0)	38 (20.8)	92 (50.3)
K5	Evidence-based medicine improves clinical management by using evidence from meta- analysis only.	59 (32.2)	48 (26.2)	76 (41.5)
K6	Evidence-based medicine does not help to promote self-directed learning.	6 (3.3)	27 (14.8)	150 (82)
K7	Meta-analysis is superior to case-control studies in evidence- based medicine.	115 (62.8)	56 (30.6)	12 (6.6)
K8	Four essential components structured in the PICO format (Patient or problem, Intervention, Comparison, Outcome) will make a good clinical question.	145 (79.2)	37 (20.2)	1 (0.5)
K9	Evidence-based medicine improves clinicians’ understanding on research methodology.	170 (92.9)	11 (6.0)	2 (1.1)
K10	Clinicians who practice evidence-based medicine become less critical in using data in systemic reviews.	15 (8.2)	67 (36.6)	101 (55.2)
K11	Evidence based medicine can be practised in situations where there is doubt about any aspect of clinical management.	166 (90.7)	15 (8.2)	2 (1.1)
K12	Improving access to summaries of evidence is appropriate to encourage evidence-based practice.	165 (90.2)	14 (7.7)	4 (2.2)
K13	Increasing number of systematic reviews, which are applicable to general practice can be found in Cochrane Library.	110 (60.1)	68 (37.2)	5 (2.7)
K14	Difficulty in understanding statistical terms is the major setback in applying evidence- based medicine.	132 (72.1)	41 (22.4)	10 (5.5)
K15	Application of evidence-based practice is cost-effective to healthcare system.	116 (63.4)	46 (25.1)	21 (11.5)

Note: Description is based on Noor Evidence-Based Medicine Questionnaire [15].

**Table A2:** Attitude toward Evidence-Based Medicine.

Item	Description	Strongly Agree n (%)	Agree n (%)	Neutral n (%)	Disagree n (%)	Strongly Disagree n (%)
A1	I believe that evidence-based medicine is a threat to good clinical practice.	115 (62.8)	64 (35.0)	3 (1.6)	1 (0.5)	-
A2	I believe practicing evidence-based medicine improves patient health outcome.	100 (54.6)	75 (41.0)	7 (3.8)	1 (0.5)	-
A3	I am keen to learn evidence-based medicine if given the opportunity.	103 (56.3)	71 (38.8)	9 (4.9)	-	-
A4	I am ready to practice evidence-based medicine in my work.	89 (48.6)	76 (41.5)	16 (8.7)	1 (0.5)	1 (0.5)
A5	I feel that research findings are very important in my day-to-day management of patients.	95 (51.9)	62 (33.9)	24 (13.1)	1 (0.5)	1 (0.5)
A6	I feel that evidence-based medicine is of limited value in emergency medicine because management in emergency care requires less scientific evidence.	6 (3.3)	17 (9.3)	35 (19.1)	84 (45.9)	41 (22.4)
A7	I believe that years of clinical experience is more valuable than evidence-based medicine.	8 (4.4)	33 (18.0)	78 (42.6)	50 (27.3)	14 (7.7)
A8	I am convinced that applying evidence-based medicine in clinical practice increases the effectiveness of my work.	62 (33.9)	103 (56.3)	18 (9.8)	-	-
A9	I feel confident managing patients with evidence-based medicine.	71 (38.8)	85 (46.4)	24 (13.1)	1 (0.5)	2 (1.1)
A10	I am certain that understanding basic mechanism of disease is sufficient for good clinical practice.	39 (21.3)	53 (29.0)	47 (25.7)	33 (18.0)	11 (6.0)
A11	I feel that access to databases is vital in obtaining journals on evidence-based medicine.	84 (45.9)	82 (44.8)	13 (7.1)	4 (2.2)	-
A12	I feel that reading the conclusions of a systemic review is adequate for clinical practice.	12 (6.6)	30 (16.4)	56 (30.6)	68 (37.2)	17 (9.3)
A13	I feel that practicing evidence-based medicine would produce better health practitioners.	75 (41.0)	91 (49.7)	15 (8.2)	-	2 (1.1)
A14	I often feel burdened whenever needing to use evidence-based medicine in practice.	9 (4.9)	36 (19.7)	74 (40.4)	56 (30.6)	8 (4.4)
A15	I think it is mandatory for physicians to continuously update their knowledge in order to deliver efficient patient care.	114 (62.3)	64 (35.0)	4 (2.2)	1 (0.5)	-
A16	I am interested in receiving education materials on evidence-based medicine as they relate to various topics.	106 (57.9)	68 (37.2)	9 (4.9)	-	-
A17	I think that educational interventions and incorporating formal teaching of evidence-based medicine at medical education is very important.	104 (56.8)	74 (40.4)	5 (2.7)	-	-

Note: Description is based on Noor Evidence-Based Medicine Questionnaire [15].

**Table A3:** Practice related to Evidence-Based Medicine.

Item	Description	Always n (%)	Often n (%)	Sometimes n (%)	Seldom n (%)	Never n (%)
P1	I apply evidence-based medicine in practice.	19 (10.4)	63 (34.4)	80 (43.7)	21 (11.5)	-
P2	I use multiple search engines for systemic review.	26 (14.2)	61 (33.3)	70 (38.3)	23 (12.6)	3 (1.6)
P3	I search for evidence-based medicine material from published journal only.	22 (12.0)	59 (32.2)	67 (36.6)	35 (19.1)	-
P4	I do not have enough time to study on evidence-based medicine.	18 (9.8)	55 (30.1)	82 (44.8)	24 (13.1)	4 (2.2)
P5	I cannot practice evidence-based medicine due to limitations of the management that I can offer to patients in emergency settings.	13 (7.1)	39 (21.3)	89 (48.6)	33 (18.0)	9 (4.9)
P6	I use evidence-based medicine for answering the questions in clinical setting.	18 (9.8)	67 (36.6)	72 (39.3)	23 (12.6)	3 (1.6)
P7	I join continuous medical education for update regarding evidence-based medicine.	23 (12.6)	75 (41.0)	47 (25.7)	32 (17.5)	6 (3.3)
P8	I promote evidence-based practice to my colleagues at workplace.	22 (12.0)	55 (30.1)	55 (30.1)	41 (22.4)	10 (5.5)
P9	I share knowledge on evidence-based medicine with my colleagues.	26 (14.2)	60 (32.8)	56 (30.6)	35 (19.1)	6 (3.3)
P10	I am involved in the development of clinical practice guidelines in Malaysia.	6 (3.3)	17 (9.3)	10 (5.5)	15 (8.2)	135 (73.8)
P11	I usually translate a clinical question from a form that can be answered from the literature.	6 (3.3)	25 (13.7)	51 (27.9)	51 (27.9)	50 (27.3)

Note: Description is based on Noor Evidence-Based Medicine Questionnaire [15].
